# Within brain area tractography suggests local modularity using high resolution connectomics

**DOI:** 10.1038/srep39859

**Published:** 2017-01-05

**Authors:** Peter N. Taylor, Yujiang Wang, Marcus Kaiser

**Affiliations:** 1 Institute of Neuroscience, Newcastle University, United Kingdom; 2 Interdisciplinary Computing and Complex BioSystems (ICOS) research group, School of Computing Science, Newcastle University, United Kingdom; 3 Institute of Neurology, University College London, United Kingdom

## Abstract

Previous structural brain connectivity studies have mainly focussed on the macroscopic scale of around 1,000 or fewer brain areas (network nodes). However, it has recently been demonstrated that high resolution structural connectomes of around 50,000 nodes can be generated reproducibly. In this study, we infer high resolution brain connectivity matrices using diffusion imaging data from the Human Connectome Project. With such high resolution we are able to analyse networks *within* brain areas in a single subject. We show that the global network has a scale invariant topological organisation, which means there is a hierarchical organisation of the modular architecture. Specifically, modules within brain areas are spatially localised. We find that long range connections terminate *between* specific modules, whilst short range connections via highly curved association fibers terminate *within* modules. We suggest that spatial locations of white matter modules overlap with cytoarchitecturally distinct grey matter areas and may serve as the structural basis for function specialisation within brain areas. Future studies might elucidate how brain diseases change this modular architecture within brain areas.

Structural connectomics, the study of structural brain connectivity, has led to the discoveries that global brain networks are small world, highly efficient, and robust to multiple failures^1^,^2^,^3^,^4^. Connectomics has proven useful in understanding brain health^5^, disease^6^, and development^7^. However, most previous whole-brain studies have focussed on the macroscopic scale, with network nodes typically numbering 100 to 1000. Only few structural connectomics studies have analysed networks of higher resolution (e.g. refs 8, 9). This means that, with a lack of higher spatial resolution, previous studies have largely been limited to analysis of global network properties, rather than localised regions (e.g. within brain areas). Furthermore, because of this, it has been difficult to investigate mappings between spatial and topological scales (e.g. measures of network scale-invariance). In addition, atlas-based low-resolution parcellations have been shown to be heavily biased by nodal properties which may not necessarily reflect network differences^10^ suggesting atlas based parcellation approaches may be sub-optimal for some situations.

One of the reasons most previous analyses have been at the macroscopic scale of parcellating the brain into around 100 nodes is that the reproducibility of higher resolution networks had not been demonstrated. However, excellent inter-acquisition agreement of ten 58,880 node surface based networks generated from ten separate scans of the same subject was recently shown^11^. Besson *et al*. showed network properties were highly and significantly correlated between scans, even when using standard 2 mm isovoxel, 64 direction diffusion MRI data. Other studies have also demonstrated the utility of high resolution approaches. For example, Calamante *et al*.^12^ analysed white matter architecture at subvoxel resolution which was later experimentally validated against *ex-vivo* mouse brain tissue^13^. Bonilha *et al*.^14^ used individual grey matter voxels as streamline termination points to estimate the density of connections, whilst Irimia *et al*.^15^ used networks of up to 50,000 nodes in their work. In addition, advances in imaging protocols mean improvements in image resolution and quality have been made. For example, the human connectome project contains diffusion MRI at 1.25 mm isovoxel^16^,^17^ which is higher than typically acquired. Finally, improvements in processing pipelines have led to better cortical segmentation (and hence more accurate surface generation^18^), and tract accuracy^19^.

A second reason most previous studies focus on low resolution networks is potential reliability (as opposed to reproducibility) issues - i.e. are inferred networks reliably capturing the actual networks? In some regards this question of reliability applies to all DW-MRI studies. For example, even the most basic question of connection direction is unknown, irrespective of resolution, which can have important implications on results^20^. Other issues and biases are also known to exist with DW-MRI tractography including potential gyral biases^21^,^22^. Despite questions of reliability, DW-MRI based connectomics has been shown to be useful in various situations^5^,^6^,^7^.

Here we attempt a pioneering investigation using high resolution connectomics to investigate 1) measures of scale-invariance, and 2) localised properties of brain areas in a single subject. Both of these two properties can only be investigated using networks of sufficiently high resolution. We demonstrate that this approach may have potential utility for analysing local properties of subsets of brain networks whilst acknowledging potential biases at such high resolution (see discussion for further details).

## Methods

Diffusion and T1w data were downloaded from the Human Connectome Project (HCP) website (ConnectomeDB). We used five representative subjects from the 500-subject release in June 2014. (The subject IDs are: 100307, 103414, 105115, 110411, 111312). All subjects are between 22 and 35 years old. In the main results of this paper we show data for the first subject, (id:100307) a 25–30 year old female, unless otherwise stated.

### Network construction

We used the preprocessed data provided by the Human Connectome Project which involves two main processing pipelines. Briefly, the PreFreeSurfer pipeline takes the T1, T2 weighted scans (0.7 mm isotropic resolution) and field map, applies gradient current distortion and then aligns the T1 and T2 scans and performs brain extraction. The gradient nonlinearity distortion correction leads to a noticeable improvement in segmentation compared to standard processing (see e.g. Fig. 10 in ref. 18). Following completion, the next FreeSurfer pipeline is run. The majority of this pipeline uses the FreeSurfer recon-all function with downsampled 1 mm isotropic T1 and T2 MRI, however, at various stages the standard recon-all process is interrupted and the higher (0.7 mm isotropic resolution) images used instead, this leads to particular improvements in white matter surface generation and pial surface registration (see e.g. Figs 13 & 14 in ref. 18). For extensive details on these pipelines we refer the reader to ref. 18 and references therein.

We downsampled the pial surface files (ID_3T_Structural_preproc_extended/ID/T1w/ID/surf/*h.pial.surf) preprocessed from the HCP data available at https://db.humanconnectome.org using the MatLab software package “Iso2Mesh”^23^ by 90%. The resulting mesh has around 50,000 triangles (nodes in the network) with a mean surface area of 3.25 mm^2^ (see Supplementary Figure S1). Downsampling has the benefit of making the subsequent network analysis less computationally intensive whilst still preserving key aspects of cortical gyrification and ensuring all triangles are more similarly sized, rather than vast differences in surface area as in the original FreeSurfer generated surface mesh which may bias connectivity to be higher in larger areas^6^. For the diffusion data we used the “DSI Studio” software package. For fiber orientation reconstruction we used generalised q-sampling imaging^19^ with a diffusion sampling length ratio of 1.25. We used a 8-fold orientation distribution function (ODF) tessellation, with 5 peaks of the ODF. For the fibre tracking step we seeded in the whole brain and tracts were terminated when the quantitative anisotropy of the voxel through which the streamline entered was below 0.6*(Otsu’s threshold). Otsu’s threshold is calculated to give the optimal separation threshold that maximizes the variance between the background and foreground^24^. We choose deterministic tractography due to the likelihood of it generating fewer false positive connections than probabilistic approaches. This is crucially important because false positives have been shown to be much more detrimental to the correct calculation of network measures than false negatives^25^. These are the preferred and recommended settings based on previous work^19^ (although see discussion for limitations of choice in parameters). Streamlines with implausible lengths (>300 mm and <10 mm) and extreme turning angles (> 60 degrees) were excluded. Other parameters were as follows, step size: 0.625 mm. Smoothing: 0. Seed orientation: primary. Seed position: subvoxel. Randomize seeding: off. Direction interpolation: trilinear. Tracking algorithm: Streamline (Euler). Inspired by previous studies^1^,^5^, only streamlines with both endpoints terminating within the grey matter were included for connection matrix generation. Grey matter areas were inserted and combined from the ‘aparc+aseg.mgz’ file downloaded from ConnectomeDB using DSI Studio. The registration quality of the grey matter ROIs was inspected and confirmed visually. A threshold of 10,000,000 streamlines was set and saved.

**Figure 1:**
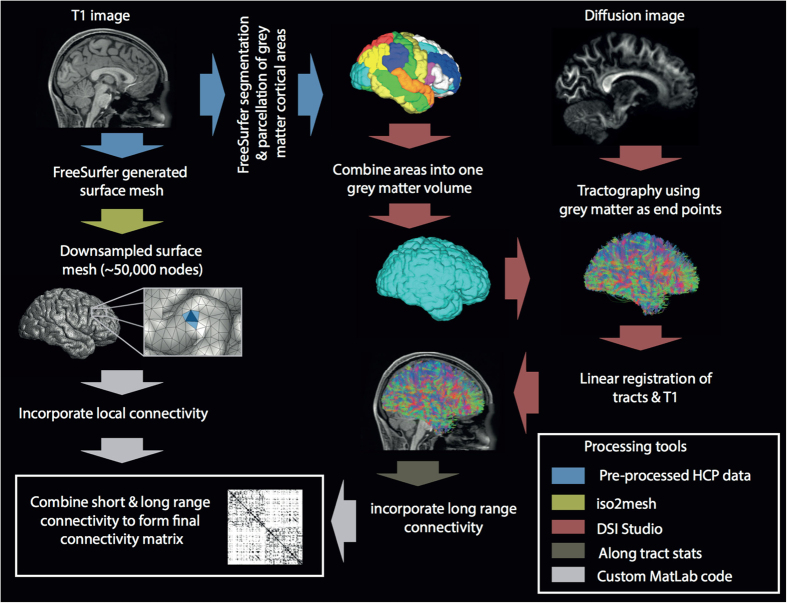
Overall procedure. Generation of the connectivity involves several steps including processing of T1w image data (left and center columns) and diffusion data (right column). The final binarised connectivity matrix has around 50,000 nodes and >1,000,000 edges.

Finally, we imported both surfaces and streamlines into MatLab^26^ and confirmed all coordinates were in the same space. Triangles on the surface mesh represent nodes in the network and are connected to their three local neighbors on each side to represent local lateral connectivity not captured by diffusion imaging. This ensures the network is fully connected (necessary for the MEMB algorithm - see next section for details) and serves as a first approximation to local grey matter connectivity with exponential decays in strength used extensively in the neural field literature^27^,^28^,^29^, discussed experimentally in ref. 30 and similarly adopted in other connectivity studies^31^,^32^. We acknowledge that local grey matter connectivity is indeed far more complex and spatially variant, however, by fixing local connectivity in this way we can ensure that our results are reflective of the experimentally derived (MRI based) data. Since the number of local connections is the same for all network nodes this does not impact any conclusions of this study. We incorporate long range connectivity by cycling through each streamline and considering a connection present between the center of the closest triangle (Euclidean distance) to the two endpoints of the streamline. The two matrices (local and long range) are then summed and binarised for analysis. A summary of the network construction and processing steps is provided in Fig. 1.

### Graph theoretic analysis

To investigate scale-invariance of the network, we used the maximum excluded mass burning (MEMB) box covering algorithm implemented in C^33^ for box sizes (1 < *ℓ*_*B*_ < 11). The algorithm computes the minimum number of ‘boxes’ required to cover the network where the maximum path length within a box is the parameter *ℓ*_*B*_.

To detect modules within brain areas we used the Louvain algorithm implemented in the brain connectivity toolbox^34^. This was done by scanning the *γ* parameter between 0.6 and 1.4 with increments of 0.02 for the subset of the cortical network corresponding to the nodes of interest 25 times to produce a modularity 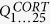. This was also repeated for a random network with the same number of nodes and edges (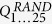). The average modularity (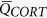 and 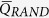) was then taken as the mean across the 25 runs. By defining *Q*_*MAX*_ as the difference between 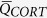 and 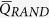, we obtain a value corresponding to each of the values of *γ*. Using the value of *γ* corresponding to the largest value of *Q*_*MAX*_ we take the most common consensus module membership of each node^35^. This approach tends to the most consistent modules at maximal modularity, relative to random networks, being detected.

## Results

### Scaling of the high-resolution structural connectome

#### Spatial scaling

**Figure 2:**
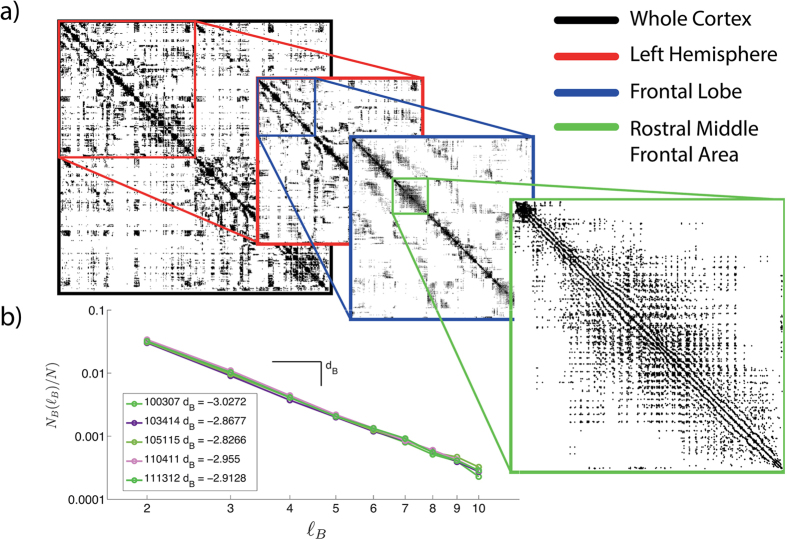
Scaling properties of the high-resolution network. (**a**) Spatial scaling: Successive zooming of the connectivity matrix. Nodes are ordered according to FreeSurfer cortical areas with the left hemisphere first. (**b**) Topological scaling: Log-log plot of *N*_*B*_(*ℓ*_*B*_)/*N* versus box size *ℓ*_*B*_ showing evidence of scale-invariance in the networks of five subjects.

Figure 2 and Supplementary movie 1 show incremental enhancements in visualisation of the network at different levels of scale. Effectively the figure ‘zooms in’ to enable easier visualisation of smaller structures, starting at the whole (>50,000 node) connection matrix (top left - bounded black matrix). The second level of zoom (red box) shows only connections within the left hemisphere, with subsequent panels showing further refinement ultimately showing connectivity within the rostral middle frontal area (green box). Within an area nodes are sorted from posterior to anterior. The rostral middle frontal area is calculated according to the Desikan-Killany (DK) atlas^36^ determined using FreeSurfer. We use the term ‘area’ to refer to a FreeSurfer (DK) area.

#### Topological scaling

This high-resolution visual representation of the network is highly reminiscent of scale-invariance. To test the hypothesis that the network topology itself is scale-invariant, rather than just our visualisation of it, we used the box covering algorithm^37^. This algorithm divides the network into non-overlapping ‘boxes’, where the size of the box is a parameter (*ℓ*_*B*_) which sets the maximum shortest path length within the box. The network is deemed scale-invariant if the minimum number of boxes required (*N*_*B*_(*ℓ*_*B*_)) to cover the whole network scales linearly with the box size on a log-log plot (i.e. follows a power law). Figure 2b shows this relationship and indeed the network is scale-invariant, observing a straight line fit. Furthermore, the gradient of the line (*d*_*B*_) is the fractal dimension - in this case approximately 2.9 for all subjects.

### Modular organisation

#### Connections between brain areas

In addition to being scale-invariant, previous studies have demonstrated the human brain network to be hierarchical and modular. We now turn our attention to modularity *within* and *between* brain areas.

**Figure 3:**
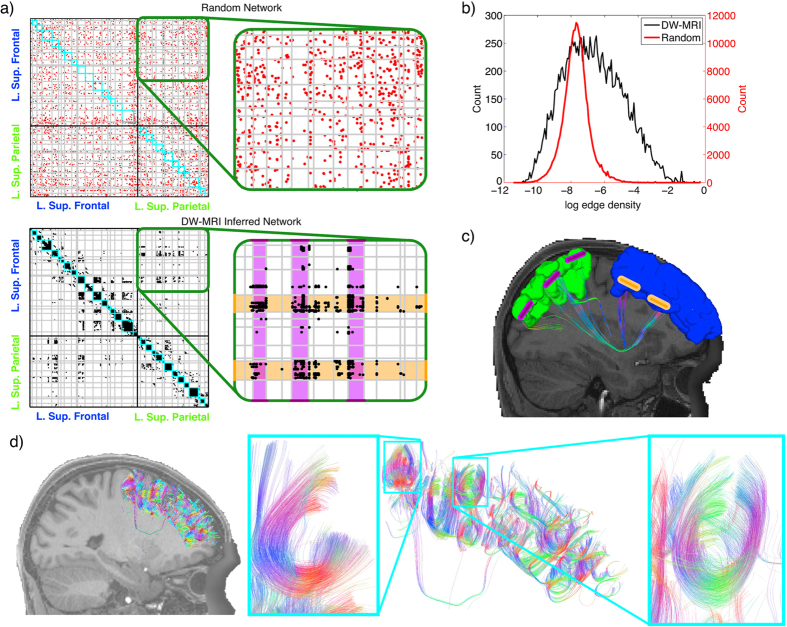
Endpoints of long range connections. (**a**) Subset of the high resolution adjacency matrix showing connectivity within and between superior frontal and superior parietal areas in the left hemisphere for random (top panel) and human (bottom panel) networks. Cyan squares indicate within-area modules from the diffusion weighted imaging (DW-MRI) inferred network. Grey lines indicate extensions of the cyan squares and make many quadrilaterals between areas. (**b**) Histogram of the non-zero edge densities in the quadrilaterals (note the different scales on the y axis and log scale on the x axis). In the human network there are fewer quadrilaterals where the edge density >0, but those that are >0 have a higher edge density than in the random networks indicating evidence for long range connections terminating in specific modules, rather than random dispersion. (**c**) An example of the module termination. Two modules (orange bars) in the superior frontal cortex (blue) are connected more than other modules to the superior parietal area, also highlighted in orange in (**a**). Bars are for illustrative purposes only, for actual modules see Fig. 4a. (**d**) Connectivity within the superior frontal area. Within the area, short association fibers dominate modules (highlighted in cyan).

As an example we show a subset of the connectivity matrix with connectivity between superior frontal (SF) and superior parietal (SP) areas in the left hemisphere. Nodes are sorted according to maximised modularity Q^38^ *within* an area, shown in Fig. 3a (bottom panel). Long range connections typically terminate in two specific modules (bottom right panel of Fig. 3a) in the SF area (highlighted in orange). This is in contrast to the random network (with the same *N, k*) where connections are more dispersed (e.g. as in the top right panel of Fig. 3a). The spatial locations of these terminations are shown in Fig. 3c.

To quantify this difference in endpoint termination between areas, we calculate the edge density of each module-to-module connection (i.e. the edge density of each grey quadrilateral in Fig. 3a between areas) for the cortical network and for five random networks. The edge density indicates the proportion of existing edges relative to all possible existing edges; i.e. the edge density is 1 if all nodes are connected to all other nodes and 0 if there are no connections between any nodes. There is a total of 350,319 module-to-module connections (based on the 845 cyan colored modules) in the full network. Each of these 350,319 grey quadrilaterals has an edge density, which we plot in the histogram in Fig. 3b. If long range connections are targeted to specific modules, there are many quadrilaterals with an edge density of zero and few with a high edge density. Conversely, if connections are randomly dispersed, as in the upper panel of Fig. 3a, there are few quadrilaterals with an edge density of zero and many with a low edge density.

Figure 3b shows the distributions of nonzero edge densities for the cortical and random networks. Indeed, connections terminate in only a small subset of all potential target modules in the human network, when compared to the random network. Furthermore, when a long range connection is present in a quadrilateral, the edge density of that quadrilateral is often several of orders of magnitude higher in the human network as compared to the random network (note the log scale on the x-axis). The significant difference in the y-axis for the random and human networks further shows that the random networks are much more dispersed and thus have significantly more nonzero edge densities. Furthermore, those targeted modules tend to be physically closer than those which are not targeted (Supplementary Figure S2), but not related to the gyral/sulcal location of the module (Supplementary Figure S3).

#### Modules within brain areas

Overall, each brain area contains at least 5 modules. In some cases, this can simply be explained by an area containing multiple Brodmann areas where each of those areas forms a module. However, many areas consist of only one Brodmann area or a number of areas that is smaller than the number of detected modules indicating that a modular organisation within-areas exists.

**Figure 4:**
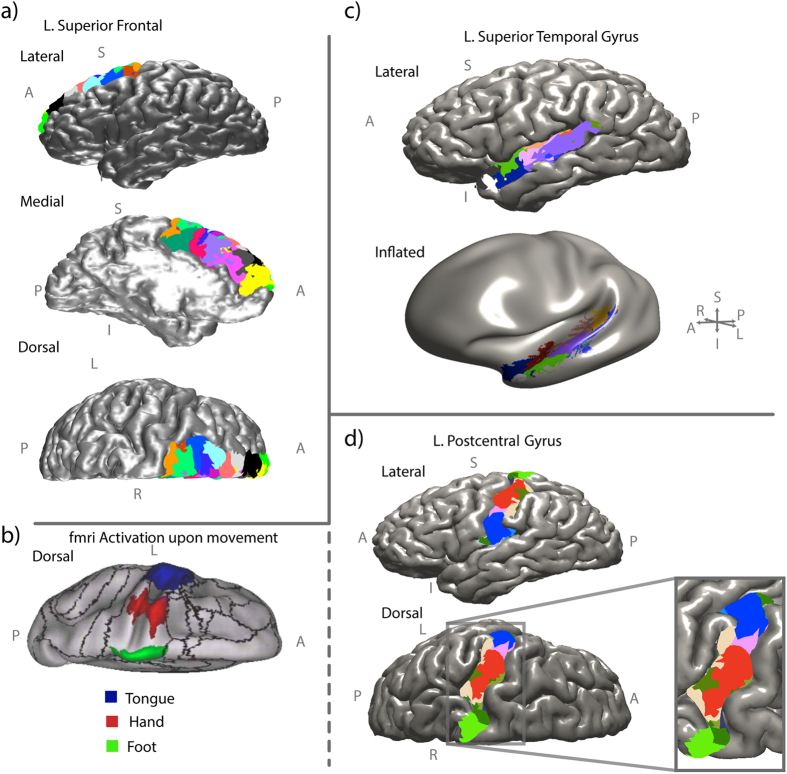
Modules are spatially localised. Nodes (surface triangles - perimeter of triangle not shown) in the network are color coded according to their module membership. Modules tend to be arranged in spatially localised areas. (**a**) Modules in the superior frontal cortex (**c**) superior temporal gryus and (**d**) the postcentral gyrus are shown from various angles to aid visualisation. The inset zoom is rotated slightly laterally for clarity. In (**b**) we show fMRI activation maps of different areas in the pre- and postcentral gyrus according to movement of different body parts^42^. Note that the functional units are comparable in size and location to structural post-central gyrus units based on high resolution connectivity shown in (**c**).

Considering the internal organisation of areas, we find that within-module connections tend to be predominantly short, highly curved association fibers (Fig. 3d). This is in agreement with previous studies^39^. To provide further evidence of the importance of short range within module connections we show color coded node module membership in the SF (Fig. 4a) and two other well studied areas. In Fig. 4c we show module membership for nodes within the superior temporal gyrus including primary auditory cortex. Some modules are located within sulci, in agreement with previous studies of cyto-architectonic grey matter structure in that area^40^,^41^. Figure 4d shows module membership for the nodes within the left postcentral gyrus, involved in sensory function. Notice how the modules tend to be arranged laterally. This is in agreement with experimental evidence suggesting different sensory functions for different body areas with distance from the midline^42^. For example, the tongue, hand and foot somatosensory areas overlap with the modules colored in blue, red and green respectively (Fig. 4b, adapted from ref. 42).

## Discussion

In this preliminary study we have built on earlier work in a single subject, which demonstrates that high resolution connectomes are reproducible^11^. We found that the network is scale-invariant across topological scales. When zooming in to the spatial scale of one area we find modules which have high intraconnectivity and are spatially localised. Finally, we found that modules tend to connect to other specific modules (rather than being distributed in their connectivity).

### Reproducibility of the reconstructed networks

Although previous studies have demonstrated that high resolution networks can be generated reproducibly^11^, it could be considered a drawback in this study that this is not demonstrated here (due to the unavailability of scan-rescan HCP data). However, when this pipeline was applied in a similar previous study good reliability was shown using lower quality data (larger voxel size, fewer diffusion directions) both within and between scans^43^. This gives confidence in the reproducibility of the connectivity generated here using the higher quality HCP data. Furthermore, the previous use of high resolution structural^11^,^15^, and functional^44^ connectomes set precedent for this work. The speculated overlap of structural modules with functional activations (Fig. 4) suggests the utility of this approach for investigating structure-function relationships. Although sufficient to demonstrate the utility of the approach, another limitation of our work is that our analysis is largely presented for a single subject. A future research direction will be to compare properties of high resolution structural connectomes between subjects^11^.

### Reliability of the reconstructed networks

Despite the apparent reproducibility, our results - in line with all DW-MRI based connectomics studies - should be considered with caution. This is because reproducibility does not necessarily imply reliability. For example, it has been argued that diffusion MRI based tractography may not be reliable for detecting even major tracts^45^. Furthermore, it has been shown that tractography into grey matter areas results in widespread differences in gyral, relative to sulcal, areas which are not reproduced experimentally^21^. This ‘gyral bias’^22^ is a known limitation of current techniques. Future tractography algorithms may seek to address this issue by normalising streamline numbers by the cortical volume associated with the white matter surface^22^, and by using different tractography rules with proximity to the grey matter. The use of 7T data should also reduce this bias^46^. Our study is possibly less affected by this bias since we binarise our network (unlike in, e.g. ref. 22 where streamline density is used to demonstrate such gyral bias), however the existence of this possible bias should be considered as potentially influencing our results. Other limitations inherent to all DW-MRI based connectomics studies also apply here such as the inability to infer directionality of inferred tracts, difficulties in tracking through dense white matter areas (e.g. the corpus callosum - which should not impact our within area analysis), and the bias for streamlines to favour directions without sharp turning angles (see e.g. refs 47, 48, 49).

Nontheless, tractography algorithms are rapidly improving and our chosen algorithm was recently shown to have the highest proportion of valid connections in open competition (http://www.tractometer.org/ismrm_2015_challenge/results) and accounts for crossing fibers using multi-shell diffusion data. Of course, diffusion based measures, however sophisticated, are an indirect measure of brain structure and do not directly reflect all connectivity. Our results should be considered with this in mind. Finally, a further factor which may influence the results presented here is the choice of tractography algorithm and associated parameters. Testing such a large parameter space would be infeasible for our work, hence we used default settings to aid ease of comparison to other studies (e.g. refs 43) and which have been tried, tested and validated at other resolutions^50^.

### High resolution network is scale-invariant

Previous studies have investigated *spatial* fractal properties of brain tissue using the box counting algorithm^51^,^52^. However, to our knowledge, only one study has investigated *topological* fractal properties of structural brain connectivity networks^53^. In that study the authors showed, using a network of 998 nodes, cortical connectivity to be scale-invariant. However, by having 998 nodes, only a limited number of data points could be generated - essentially to measure scale-invariance across topological scales, multiple scales need to be measured. By using a high resolution network of >50,000 nodes we are able to capture multiple spatial and topological scales and confirm the previous results of ref. 53 with greater confidence.

Self-similar properties of high-resolution functional brain connectivity networks have been studied extensively by Gallos and colleagues^54^,^55^. In those studies the authors showed that densely connected fractal modules are crucially related to functional specialization in ‘large world’ networks where pathways can contain a large number of intermediate nodes along a shortest path. Importantly, the authors also showed that long-range ‘weak tie’ connections, when added between these modules, lead to better global network integration. These ‘weak ties’ revert it to the ‘small world’ network frequently described in the literature providing, on average, a low number of intermediate steps along the path between brain regions^56^. Cortical networks therefore achieve a balance between local specialized processing and global integration through their hierarchical organization^57^. Our study extends the work by Gallos *et al*. by finding a fractal architecture at the level of structural connectivity, providing the basis for observations in functional networks.

### High resolution allows within-area network analysis

A further benefit of studying the high resolution connectome, and a key novelty of our work, is that this allows the analysis of within-area connectivity. We find modules within brain areas are spatially localised. In most previous connectomics studies, within-area connections are largely disregarded. By studying the network at this level we have uncovered the spatial organisation of within-area modularity (Fig. 4). Modularity in the network is highly beneficial as this leads to local segregation of processing^4^,^58^, a rich dynamical repertoire^59^ and dynamic functionality within modules^60^. Indeed, it has been suggested that brain connectivity networks are modular and hierarchical (e.g. fractal), however, to our knowledge this has not been reported using structural human brain connectivity at this spatial scale.

Previous studies have highlighted the existence of modules at the macroscopic scale^61^,^62^. By using high resolution data we have shown that within the primary somatosensory area further modules can be detected which we postulate correspond with functionally distinct operations such as hand, tongue and foot sensation. These nested modules within modules are in agreement with our findings of the self-similar properties of the network. We speculate that with an even higher resolution further modules exist. For example, within the hand module there may be specific modules for separate fingers. In single subjects this may prove difficult to detect, however a direction of future work could be to incorporate subject-specific functional imaging such as that obtained through fMRI.

### High resolution shows between-area module-module terminations

A further result of ours is that the between-area connections tend to connect specific modules to specific modules, rather than fanning out at either or both ends (Fig. 3b,c). It is unclear what mechanisms drive this specificity of long range targeting, however there are several potential explanations. These include common input from subcortical areas (e.g. the thalamus^63^), shared cytoarchitecture^64^, gyral preference, or shorter distance. We tested the latter two and found no preference for module-module connections to target gyri, sulci or saddles (effect size <0.1). However, we found a substantial and significant association with distance; i.e modules which are closer together are more likely to be connected (Figure S2). It is unclear how much of this is attributable to any bias of tractography to favour shorter streamlines though^65^. The existence of some long range module-module connections suggests that minimising physical distance between modules is not the only driving mechanism however (see e.g. right side of Figure S2g).

## Conclusion

To summarise, we have shown that an inferred cortical network of the human brain is scale-invariant, with a hierarchical organisation of the modular architecture. It has not escaped our notice, that the described modular architecture within brain areas suggests a potential structural mechanism for implementing distinct functions. Moving forward, it will be of interest to use these techniques to investigate localised brain dysfunction in, for example, focal epilepsy patients where spreading through local connectivity is known to be important^6^ and in larger subject cohorts^11^.

## Additional Information

**How to cite this article**: Taylor, P. N. *et al*. Within brain area tractography suggests local modularity using high resolution connectomics. *Sci. Rep.* **7**, 39859; doi: 10.1038/srep39859 (2017).

**Publisher's note:** Springer Nature remains neutral with regard to jurisdictional claims in published maps and institutional affiliations.

## Supplementary Material

Supplementary Information

Supplementary Video S1
