# Supplementary material for: Within brain area tractography suggests local modularity using high resolution connectomics
Source: Sci Rep. 2017 Jan 5;7:39859. doi: 10.1038/srep39859 (PMC5213837; doi:10.1038/srep39859)
Supplement: Supplementary Information [file srep39859-s1.doc]

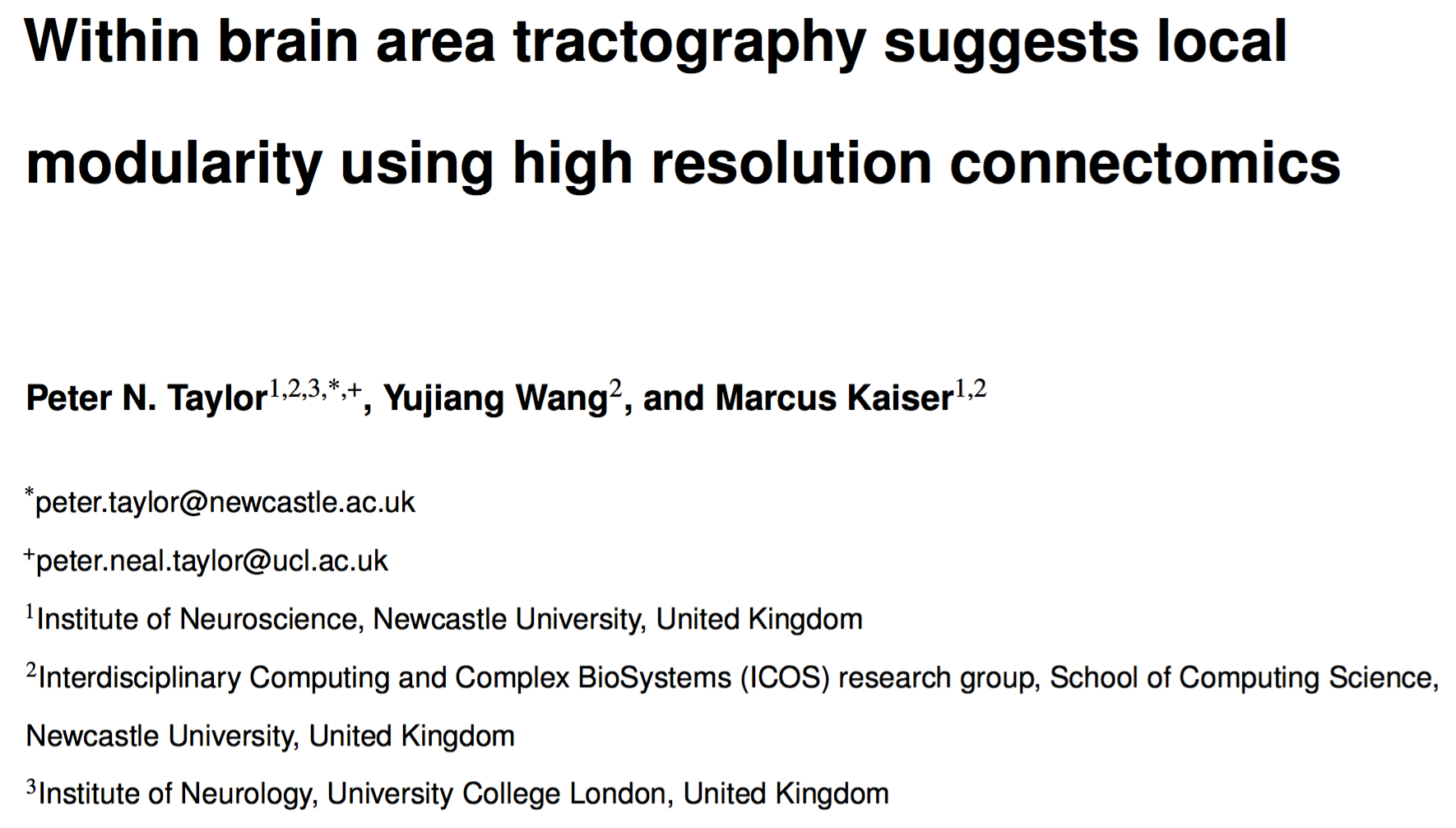


Supplementary information.


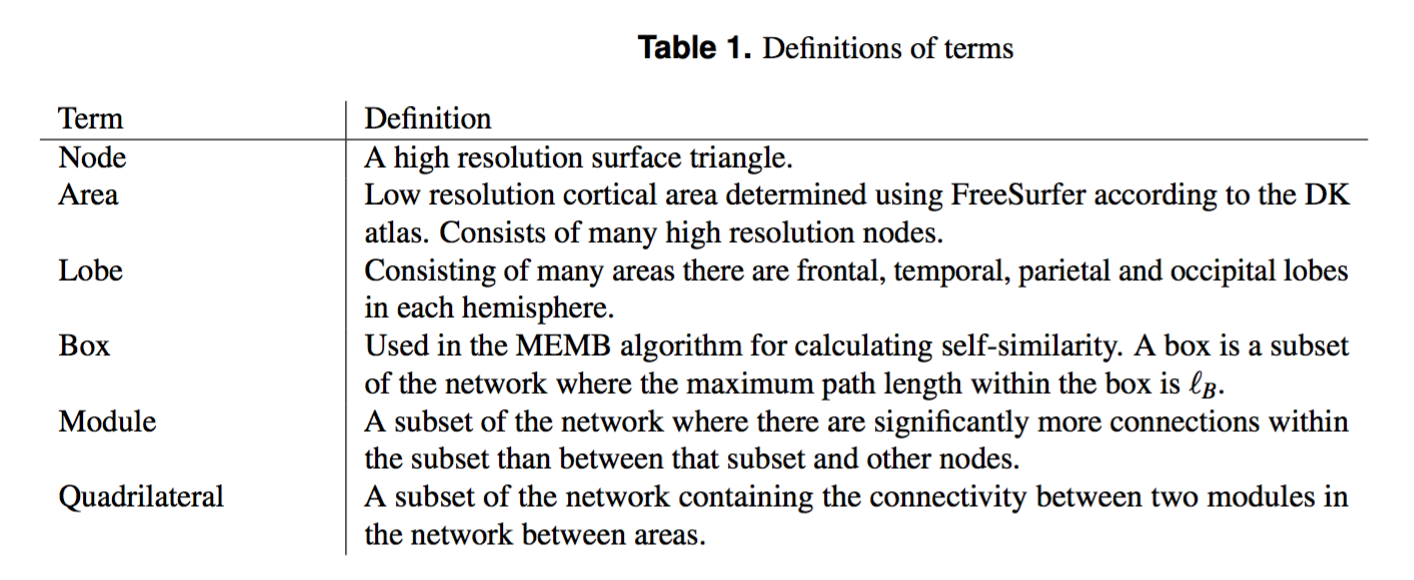


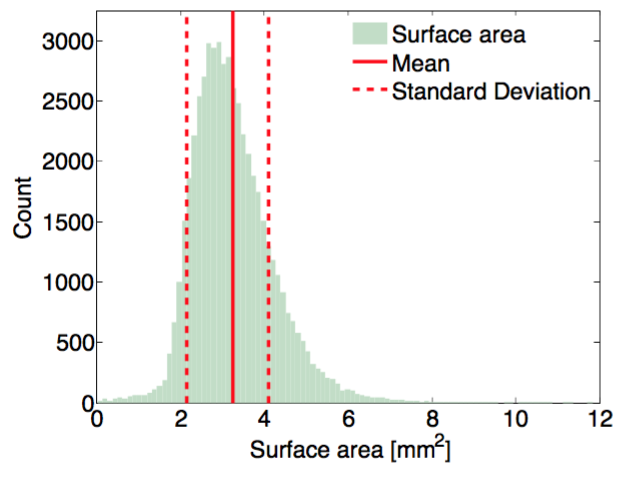


**Supplementary Figure S1.** Histogram of node surface area Solid and dashed lines indicate mean and one standard deviation. Over 95% of nodes’ surface area are within two standard deviations of the mean.


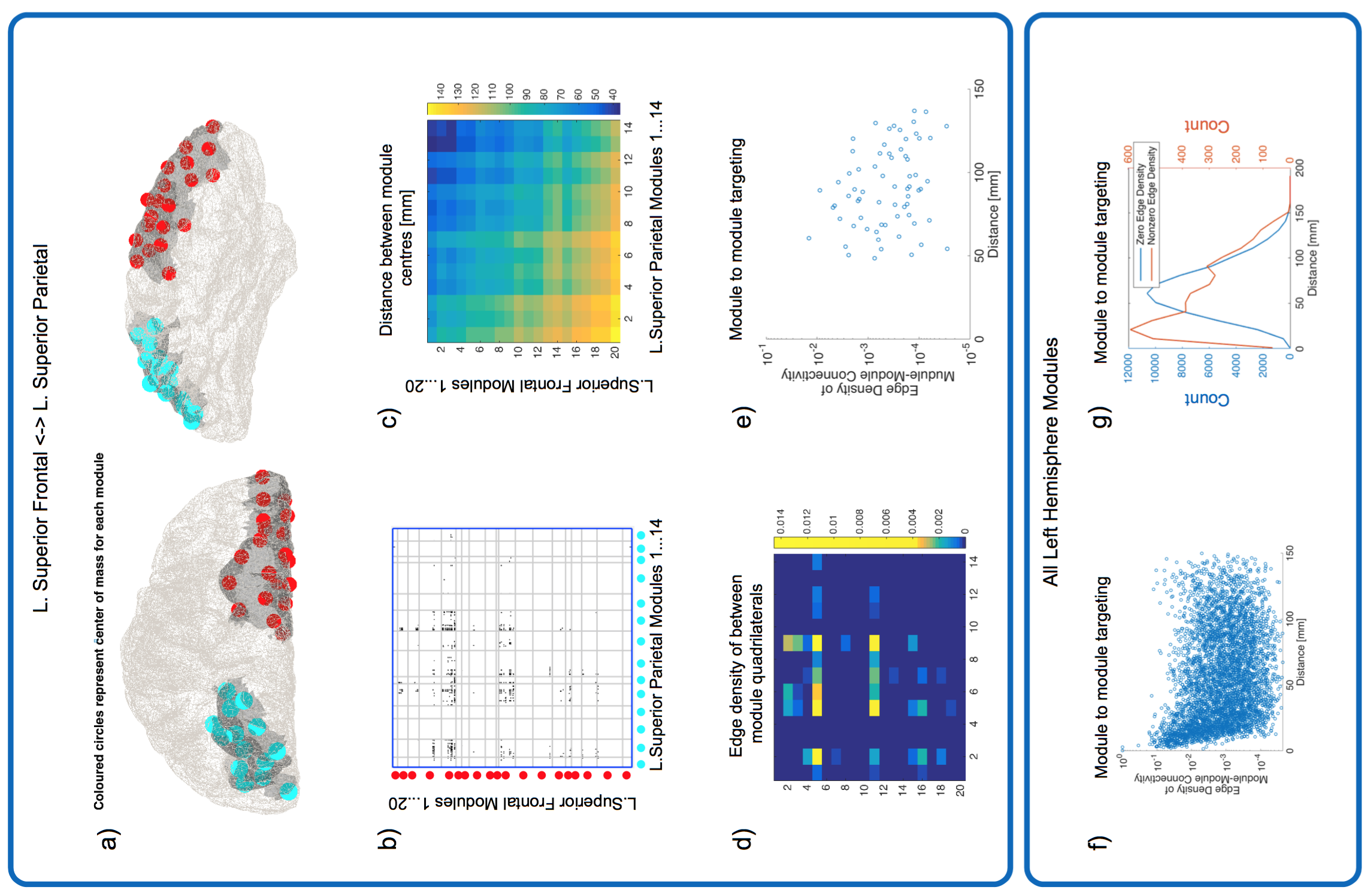


**Supplementary Figure S2: Between area module targets tend to be closer in space than by chance. a)** Within area modules’ center of mass (median of x,y,z coordinates). **b)** Subset of the high resolution connectivity matrix showing only connections between L.Sup.Par and L.Sup.Front areas. **c)** Euclidean distance between the module centers shown in a). **d)** Edge density of each of the potential module-module target quaderilaterals (inferred from panel b). **e)** Scatter plot of the nonzero values in panel d) against those corresponding values in panel c). **f)** Repeat of panel e) but for all between area module-module connections. **g)** Histogram of distances between module centers where a connection does/does not exist between modules (red/blue). Where a connection exists those modules tend to be closer in space (p<<0.01, effect size = 0.34).


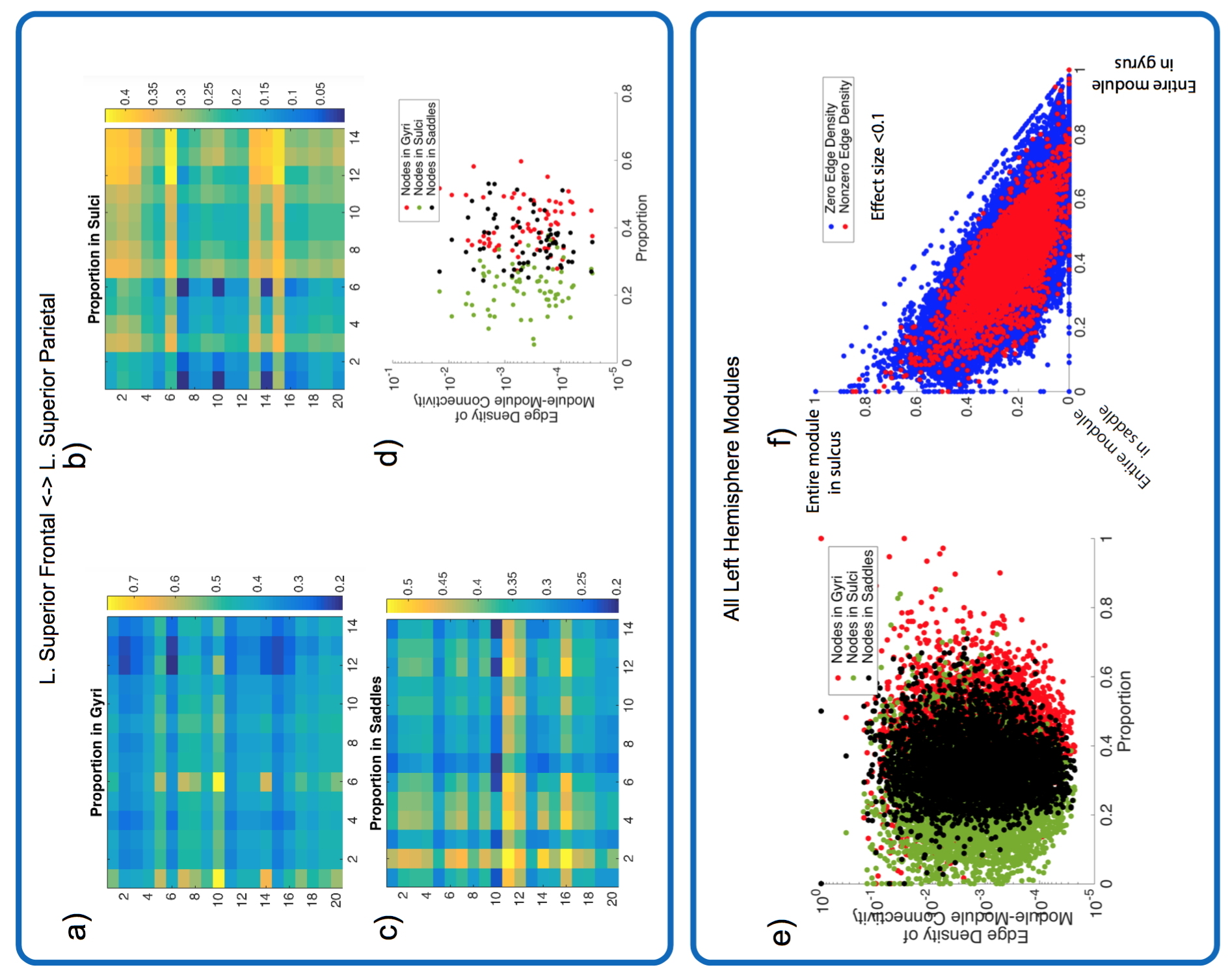


**Supplementary Figure S3: Between area module targets do not tend to favour gyral crowns.** Each module in the high resolution network is composed of multiple nodes. Here we show what proportion of nodes within the two modules on the x, and y axes are located in gyri (**a)**, sulci (**b)** and saddles (**c)**. The matrices in (a,b,c) therefore sum to 1. If there were a tendency for gyri modules to target gyri modules then panel a) would have a high similarity to figure S2d. **d)** No correlation between proportion of nodes in gyri, sulci or saddles with edge density for L.SupPar module – L.SupFront connections **e)** Repeat of panel d) but for all between area module-module connections in the left hemisphere. **f)** All potential between area module-module connections in the left hemisphere. Each dot represents a quadrilateral. Red (blue) dots indicate those quadrilaterals where there does (does not) exist a connection between modules. There is no substantial difference between the two distributions (Cohen’s D effect size <0.1) suggesting no preference for between area module-module targets.

**Supplementary Video S1** High resolution connectivity matrix shown with successive levels of zoom.
